# Tubeless uniportal thoracoscopic wedge resection with modified air leak test and chest tube drainage

**DOI:** 10.1186/s12893-020-00910-9

**Published:** 2020-11-30

**Authors:** Zhengcheng Liu, Rusong Yang, Yang Sun

**Affiliations:** 1 Department of Thoracic Surgery, Nanjing Chest Hospital, Treatment and Research Center for Pulmonary Nodule in Nanjing Medical University, Nanjing, 210029 Jiangsu China; 2 Affiliated Nanjing Brain Hospital, Nanjing Medical University, Nanjing, 210029 China; 3 Department of Anaesthesia, Nanjing Chest Hospital, Nanjing, 210029 China

**Keywords:** Tubeless, Uniportal, Thoracoscopic, Non-intubated

## Abstract

**Background:**

To investigate whether tubeless uniportal thoracoscopic wedge resection with modified air leak test and chest tube drainage has better short-term outcomes than non-intubated approach with chest tube drainage.

**Methods:**

Data were collected retrospectively from January 2017 and December 2019. Tubeless group included 55 patients with pulmonary nodules underwent tubeless uniportal thoracoscopic wedge resection, 211 patients underwent non-intubated uniportal thoracoscopic wedge resection with chest tube drainage were included in drainage group. Peri-operative outcomes between two groups were compared.

**Results:**

After 1:1 matching, 110 patients remained for analysis, baseline demographic and clinical variables were comparable between the two groups. Mean incision size was 3 cm in both group. Mean operative time was 59.3 min in tubeless group and 52.8 min in drainage group. The detectable mean lowest SpO_2_ and mean peak EtCO_2_ during operation was acceptable in both groups. Conversion to intubated ventilation or thoracotomy was not required. No patient failed the air leak test and did not undergo a tubeless procedure. Mean postoperative hospital stay was 1.5 days in tubeless group and 2.5 days in drainage group. Residual pneumothorax or subcutaneous emphysema was not frequent and mild in tubeless group. Side effects were rare and mild, including cough and hemoptysis. No re-intervention or readmission occurred. The postoperative VAS score was significantly lower in tubeless group.

**Conclusions:**

Tubeless uniportal thoracoscopic wedge resection with modified air leak test and chest tube drainage is feasible and safe for selected patients with peripheral pulmonary nodules, it might reduce post-operation pain and lead to faster recovery.

## Background

Thoracoscopic surgery is an option for diagnosis and treatment of peripheral pulmonary nodules (PPN). Non-intubated anaesthesia might prevent the adverse events caused by intubation, ventilation and extubation procedure [1]. Tubeless approach could further relieve wound pain associated with chest tube placement [2]. However, full expansion of lung sometimes could not be achieved after operation and presented as pneumothorax [3]. We describe a modified air leak test and chest tube drainage method in tubeless uniportal thoracoscopic wedge resection.

The objective is to investigate whether tubeless uniportal thoracoscopic wedge resection with modified air leak test and chest tube drainage has better short-term outcomes than non-intubated approach with chest tube drainage.

## Methods

### Patient selection

A retrospective analysis was performed, a total of 55 consecutive patients who underwent tubeless uniportal thoracoscopic wedge resection between August 2018 and December 2019 were retrospectively evaluated (Tubeless Group). Patients in both group underwent non-intubated anaesthetic. The first tubeless uniportal thoracoscopic wedge resection was performed in March 2018, the first non-intubated uniportal thoracoscopic wedge resection with chest tube drainage was performed in January 2017, and the initial 30 cases were not included due to the learning curve effect, a total of 211 patients were included in control group (Drainage Group). Consultants in our department all agreed that either technique was suitable for each patient. Cases with conversion to tracheal intubation, thoracotomy or lobectomy were excluded.

**Fig. 1:**
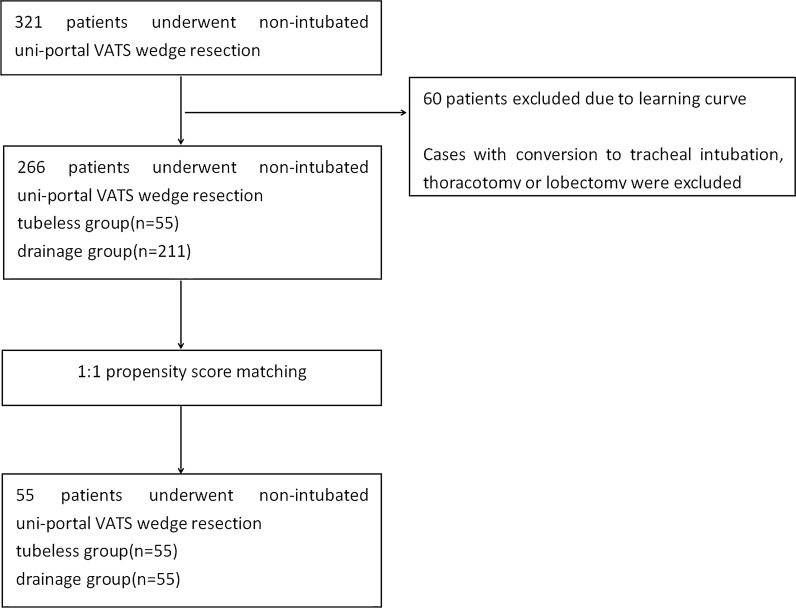
The flowchart of the study

Patients considered appropriate for this technique met the following criteria: single peripheral pulmonary nodule fit for wedge resection (pure GGOs or GGOs with a solid component, distance from visceral pleura within 2 cm), age 18–65, no cardiopulmonary dysfunction, American Society of Anaesthesiologists (ASA) grade of I–II. Patients with a bleeding disorder, sleep apnea, evidence of potential pleural adhesion, overweight (BMI > 28), and potential difficult airway for intubation were considered unsuitable (Fig. 1). The control group consisted of patients who met the same inclusion and exclusion criteria who underwent the same anesthesia and surgical procedures but with postoperative chest tube drainage.

The application of this new technique was approved by the institutional review board at Nanjing Chest Hospital (number of the ethics approval: 2017-KL002-02), all patients provided written informed consent before operation.

### Anaesthesia method

Anaesthesia protocol was described previously [4]. In brief, after intravenous infusion of dexmedetomidine 1 μg/kg by pump injection within 15 min, anesthesia was induced with intravenous dexamethasone 10.00 mg, midazolam 0.10 mg/kg and sufentanil 0.1–0.2 µg/kg, target plasma concentration of propofol 2–3 µg/ml was controlled by target-controlled infusion (TCI). Pre-lubricated laryngeal mask was inserted for spontaneous ventilation with 100% inspired oxygen (4–5 l/min) to keep oxygen saturation above 95%.

Intercostal nerve block was performed with 0. 375% ropivacaine and 1.00% lidocaine at the rib space where incision located, it was also performed at both one rib space above and one rib space below. Maintaining of anesthesia was done with TCI of propofol (target plasma concentration of 1–2 µg/ml), dexmedetomidine (0.5–1 µg/kg/h) and remifentanil (0.1–0.5 µg/kg/h). After making the incision and opening the ipsilateral pleura, a gradual and natural collapse of the lung occured during spontaneous ventilation procedures. Thoracic vagus nerve block was performed along vagus nerve beside trachea, about 1 cm above azygos vein. 5 ml of 2% lidocaine was sprayed on the lung surface under thoracoscopic guidance to help reduce cough reflex induced by thoracoscopic manipulation.

### Surgical technique for uniportal VATS

The patient was kept in lateral decubitus position. A single incision was performed at the fifth intercostal space along the anterior axillary line. During operation, a 10-mm 30-degree thoracoscope (Karl Storz) and several thoracoscopic instruments were simultaneously fitted into the uni-port. PPN was localized by preoperative CT-guided localization with hook-wire when necessary. Wedge resection was achieved using an articulating endoscopic linear cutter (Ethicon or Covidien). Lymph node sampling was performed for PPN containing invasive component (minimal invasive adenocarcinoma or invasive adenocarcinoma).

### Modified air leak test and chest tube drainage in tubeless group

**Fig. 2:**
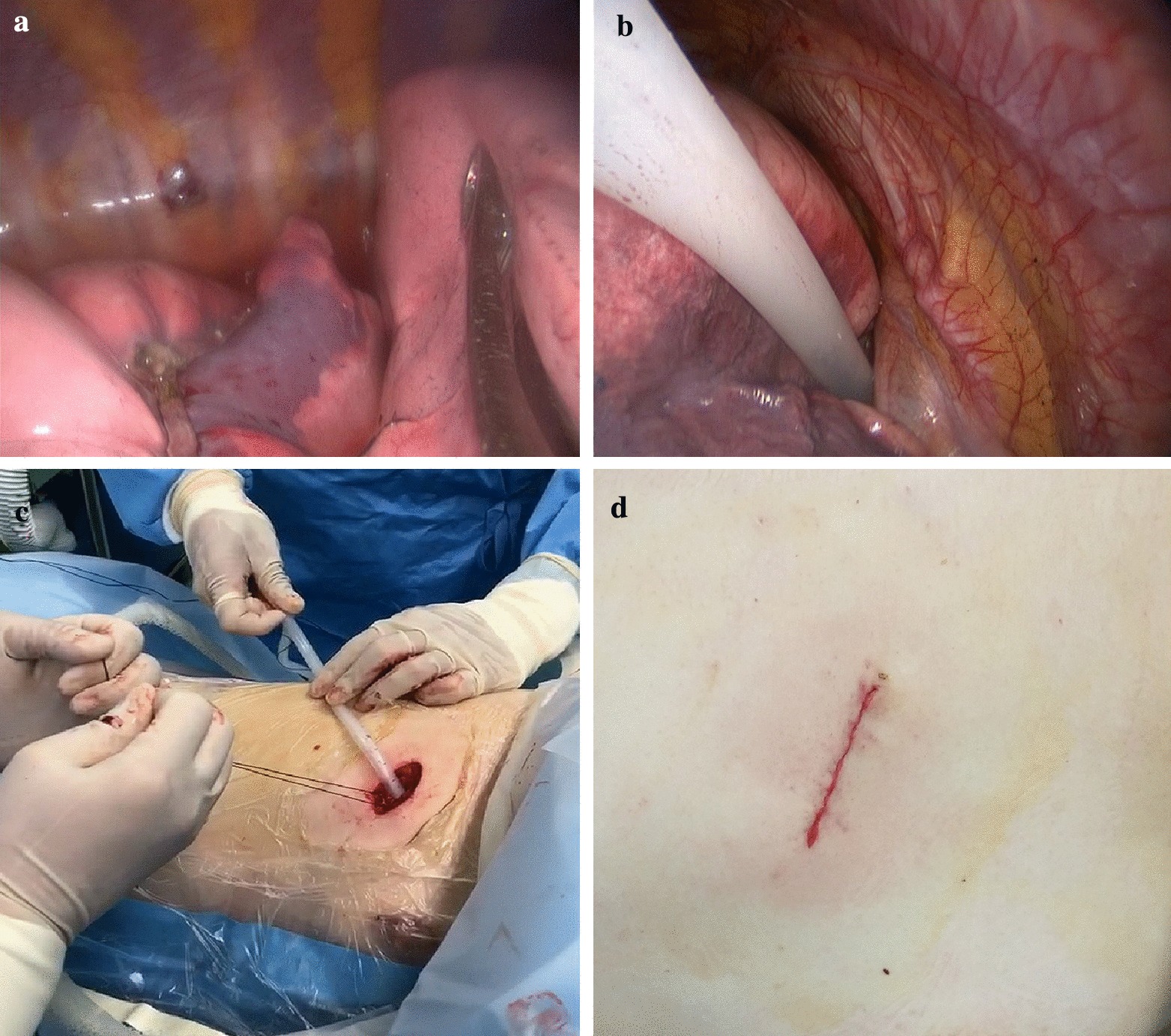
Air leak test and chest tube drainage. **a** Lung was immersed in saline and expanded for air leak test. **b** A chest tube was inserted to top of thoracic cavity. **c** Chest tube was connected to a water-sealed bottle. **d** Incision was closed with continuous sutures

At the end of operation, the lung was immersed in saline and expanded fully for air leak test, the airway pressure was up to about 20 cmH_2_O, which was assisted by hand controlled ventilation through laryngeal mask (Fig. 2a).

**Fig. 3:**
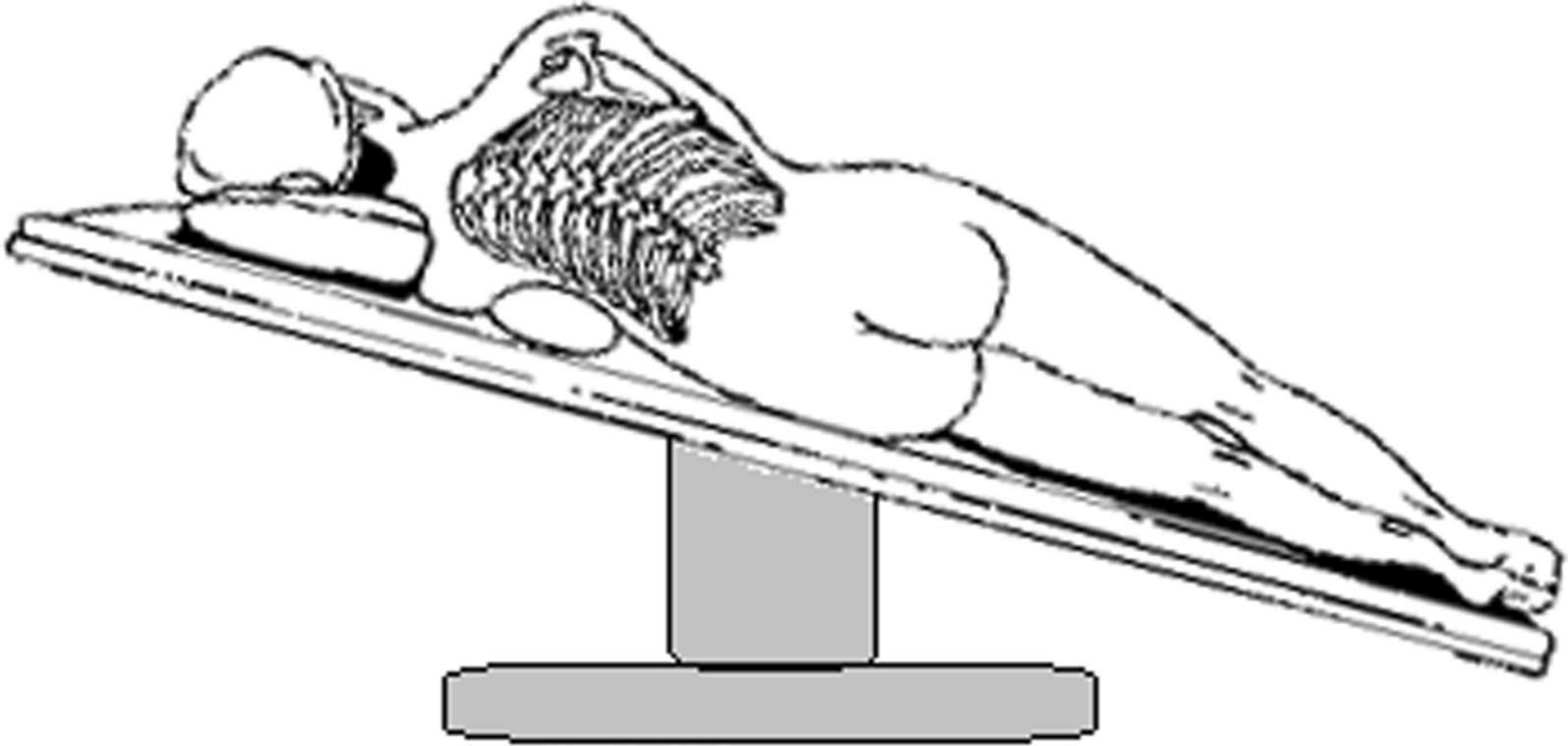
Patients was changed to reverse trendelenburg position with 30° before modified air leak test and chest tube drainage in tubeless group

After saline was sucked out, a 22–24F chest tube was inserted at the top of thoracic cavity (Fig. 2b), and patients was changed to reverse trendelenburg position with 30° (Fig. 3). Chest tube was placed at posterior one-third position of incision, serratus anterior muscle was interrupted sutured with one suture around chest tube left untied. Then chest tube was connected to a water-sealed bottle, and the lung was expanded again by hand controlled ventilation as before (Fig. 2c). When air leak excluded, chest tube was slowly removed, usually 5 cm after at least one full breath. The last suture was tied at the same time that chest tube left the thoracic cavity. Then incision was closed with continuous sutures (Fig. 2d). Chest tube drainage would be remained in patients with any air leak during this test were.

### Chest tube insertion and remove in drainage group

One 22F chest drain was inserted to thoracic cavity at the end of the operation, it was placed at posterior part of the uniportal incision. Drain removal criteria were as follows: no observed air leak and total drainage less than 200 ml in 24 h; normal chest roentgenograph; normal vital signs; good overall medical status. No patient was discharged with a chest tube in situ.

### Postoperative treatment and follow-up

**Fig. 4:**
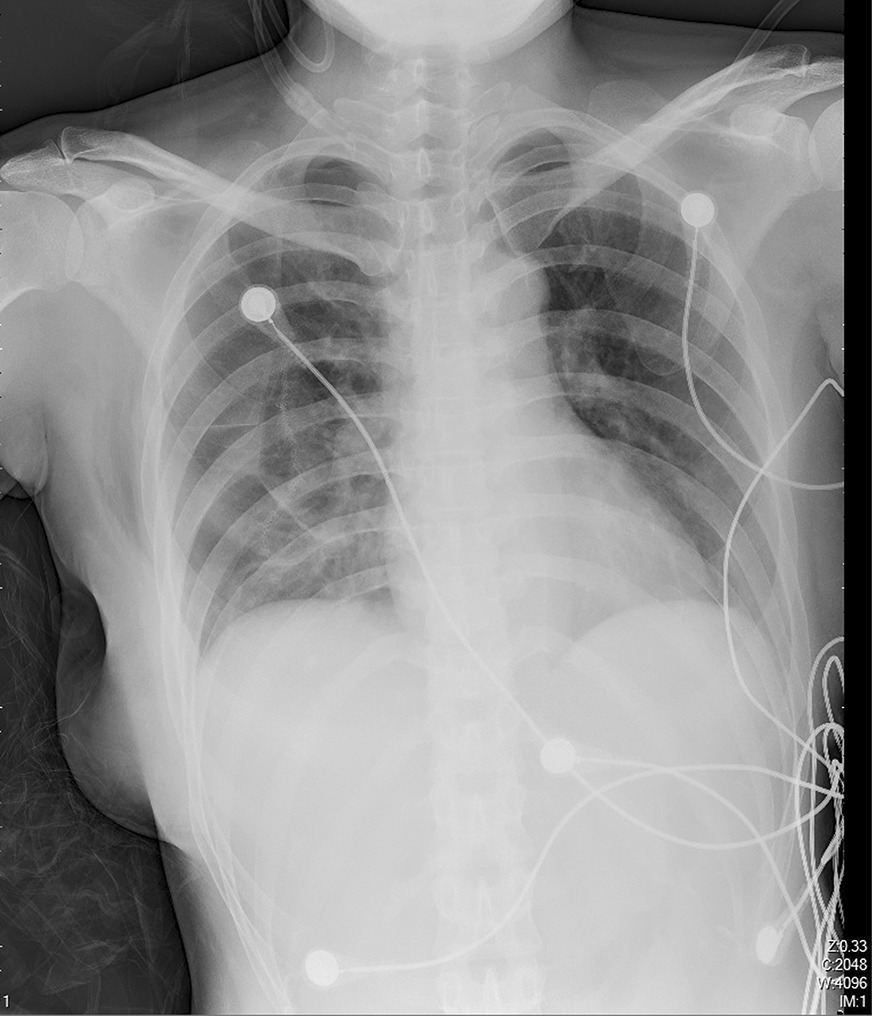
Chest radiography was performed post-operatively

Chest radiography was performed 6 h and the following morning post-operatively in tubeless group (Fig. 4), it was performed at the first day postoperatively and every 3 days until discharge in drainage group. Drinking and meal intake were resumed after bowel sounds returned with no nausea or vomiting.

The size of residual pneumothorax was defined as the largest distance between the pleural line and the chest wall on chest radiography. Intervention (Chest tube drainage or needle aspiration) should be performed when the size was larger than 3 cm. Subcutaneous emphysema was defined as the presence of subcutaneous air on chest radiography. Residual pleural effusions were defined as blunting of the costophrenic angle in the ipsilateral lung on chest radiography.

Postoperative wound pain was monitored using the Visual Analogue Scale (VAS), where 0 indicates no pain and 10 indicates the worst possible pain. VAS was evaluated on postoperative days 1, 3, 7, 30 and 60. Wound healing was evaluated by the surgeon 1-month after operation, it was graded as satisfactory, unsatisfactory, and debridement required.

### Statistical analysis

SPSS 16.0 for Windows (IBM, Armonk, NY) was used for analysis. To minimize the impact of potential confounders and selection bias, propensity score analysis was used to compensate for the differences in baseline patient characteristics between the two groups of patients. Patients in the two groups were 1:1 matched using the nearest propensity score on the logit scale. Variables that could influence the outcomes of treatment were matched, including age, gender, body mass index (BMI), ASA status class, and maximal lesion size. After PSM, differences in continuous and categoric clinical characteristics were compared.

Continuous data are presented as mean and SD and were analyzed with two-sample Student’s t tests for independent data. Categorical variables are given as a count and percentage of patients and analyzed with the χ^2^ or Fisher’s exact test. All tests were two-sided, P-values < 0.05 were considered statistically significant.

## Result

55 patients (23 males and 32 females) with a median age of 44 years (range: 22–69 years) underwent tubeless uniportal thoracoscopic wedge resection successfully. 17 patients was current or ever smokers. A total of 211 patients underwent non-intubated uni-portal thoracoscopic wedge resection with chest tube drainage were included in control group. Before matching, there were no significant differences between the two groups in terms of gender, age, smoking history, pulmonary function (FEV1), BMI, ASA score, comorbidity, maximum diameter of the lesion, nodule depth, the lung lobe where lesion located. After 1:1 matching, 110 patients remained for analysis, baseline demographic and clinical variables were comparable between the two groups (Table 1).

**Table 1:** Characteristics of patients who underwent non-intubated uni-port video-assisted thoracoscopic wedge resection with or without chest tube drainage

	Before propensity score matching			After propensity score matching		
	Tubeless group (n = 55)	Drainage group (n = 211)	P-value	Tubeless group (n = 55)	Drainage group (n = 55)	P-value
Gender			0.54			0.78
Male	23 (41.8%)	92 (43.6%)		23 (41.8%)	24 (43.6%)	
Female	32 (58.2%)	109 (56.4%)		32 (58.2%)	31 (56.4%)	
Age	44.8 ± 11.1	45.9 ± 10.8	0.36	44.8 ± 11.1	45.1 ± 10.5	0.84
Smoking history (no. of smokers) (%)	17 (30.9%)	83 (39.3%)	0.19	17 (30.9%)	19 (34.5%)	0.45
FEV1 (L)	2.49 ± 0.51	2.53 ± 0.69	0.42	2.49 ± 0.51	2.51 ± 0.41	0.73
FEV1, % of prediction	115.3 ± 12.5	112.6 ± 15.5	0.57	115.3 ± 12.5	113.4 ± 13.7	0.76
Body mass index (kg/m^2^) (median and range)	22.9 ± 2.7	23.6 ± 3.3	0.29	22.9 ± 2.7	23.2 ± 2.1	0.58
ASA status class			0.83			1.00
I	49 (89.0%)	192 (90.1%)		49 (89.0%)	49 (89.0%)	
II	6 (11.0%)	19 (9.9%)		6 (11.0%)	6 (11.0%)	
Comorbidity			0.25			0.79
Hypertension	2 (3.6%)	12 (5.6%)		2 (3.6%)	2 (3.6%)	
Diabetes mellitus	2 (3.6%)	3 (1.4%)		2 (3.6%)	1 (1.8%)	
Other	1 (1.8%)	4 (1.9%)		1 (1.8%)	2 (3.6%)	
Maximal lesion size (mm)	9.8 ± 3.3	8.8 ± 3.9	0.21	9.8 ± 3.3	9.3 ± 3.7	0.62
Nodule depth (mm)	6.3 ± 4.7	7.1 ± 5.0	0.32	6.3 ± 4.7	6.7 ± 4.5	0.43
Lesion location			0.34			0.55
Right upper lobe	15 (27.2%)	58 (27.4%)		15 (27.2%)	15 (27.3%)	
Right lower lobe	14 (25.4%)	48 (22.8%)		14 (25.4%)	12 (21.8%)	
Left upper lobe	13 (23.6%)	59 (30.0%)		13 (23.6%)	16 (29.1%)	
Left lower lobe	13 (23.6%)	46 (21.8%)		13 (23.6%)	12 (21.8%)	

*ASA* American Society of Anesthesiologists, *FEV1 *forced expiratory volume in 1 s

Table 2 illustrates postoperative data. Mean incision size was 3 cm in both group. Mean operative time was 59.3 min (35–75) in tubeless group and 52.8 min (30–75) in drainage group.

**Table 2:** Postoperative data of patients who underwent non-intubated uni-port video-assisted thoracoscopic wedge resection with or without chest tube drainage

Perioperative outcomes	Tubeless group (n = 55)	Drainage group (n = 55)	P-value
Operation time (range) (min)	59.3 ± 10.6	52.8 ± 11.4	0.16
SpO_2_ (%)	96.1 ± 2.8	96.0 ± 2.6	0.97
EtCO_2_ (mmHg)	44.9 ± 4.8	45.1 ± 5.3	0.82
Blood loss (range) (ml)	10.7 ± 6.9	10.2 ± 7.1	0.83
Preoperative CT-guided localization	50 (90.9%)	51 (92.7%)	0.92
Thoracic adhesion (%)			0.79
Adhesion	3 (5.4%)	2 (3.6%)	
No adhesion	52 (94.6%)	53 (96.4%)	
Operative method			0.11
Wedge resection only	36 (65.4%)	33 (60.0%)	
Wedge resection with lymphadenectomy	19 (34.6%)	22 (40.0%)	
Number of lymph node resection	2.1 ± 1.9	2.4 ± 2.1	0.85
Time to drink water (range) (min)	129 ± 22.5	133 ± 19.9	0.57
Drainage duration (range) (days)	N/A	1.9 ± 0.9	/
Hospital stays after surgery	1.5 ± 0.5	2.5 ± 0.8	< 0.01
Pneumothorax			0.94
No	40 (72.7%)	43 (78.2%)	
Pneumothorax (observation)	15 (27.3%)	12 (21.8%)	
Pneumothorax (intervention required)	0 (0.0%)	0 (0.0%)	
Subcutaneous emphysema			0.81
No	46 (83.6%)	47 (85.4%)	
Subcutaneous emphysema (observation)	9 (16.4%)	8 (14.6%)	
Subcutaneous emphysema (intervention required)	0 (0.0%)	0 (0.0%)	
Pleural effusion required drainage	1 (1.8%)	1 (1.8%)	1.00
Prolonged tube drainage > 3 days	0 (0.0%)	0 (0.0%)	1.00
Irritable cough	6 (10.9%)	7 (12.7%)	0.86
Postoperative hemoptysis	21 (38.2%)	24 (43.6%)	0.52
Atrial fibrillation	0 (0.0%)	0 (0.0%)	1.00
Mortality	0 (0.0%)	0 (0.0%)	1.00
VAS score (POD1)	1.0 ± 0.7	3.0 ± 0.9	< 0.01
VAS score (POD3)	0.5 ± 0.5	1.1 ± 1.5	< 0.01
VAS score (POD7)	0.4 ± 0.4	0.7 ± 0.4	0.31
VAS score (POD30)	0.2 ± 0.3	0.4 ± 0.5	0.64
VAS score (POD60)	0.1 ± 0.2	0.1 ± 0.2	1.00
Wound healing			0.15
Satisfied	54 (98.2%)	51 (92.7%)	
Unsatisfied	1 (1.8%)	4 (7.3%)	
Debridement required	0 (0%)	0 (0%)	

*POD* post-operation day

Mild adhesion was found in 3 patients in tubeless group and 2 patients in drainage group. The detectable mean lowest SpO_2_ and mean peak EtCO_2_ during operation was acceptable in both groups. Conversion to intubated ventilation or thoracotomy was not required. No patient failed the air leak test and did not undergo a tubeless procedure. Oral fluid intake was allowed about 2 h after operation in both groups. The postoperative course was uneventful. Mean postoperative hospital stay was 1.5 days (1–3) in tubeless group and 2.5 days (2–5) in drainage group.

In tubeless group, the mean diameter of the lesions was 9.8 mm (7–14). The locations of PPN included right upper lobe, right lower lobe, left upper lobe and left lower lobe. Histologic examination showed 31 cases of adenocarcinoma in situ, 17 cases of minimal invasive adenocarcinoma, 2 cases of highly differentiated invasive adenocarcinoma, 5 case of benign lesion. Lymphadenectomy was performed in 19 cases.

In tubeless group, chest radiography revealed residual pneumothorax in 15 patients 6 h after operation (11 cases of wedge resection in upper lobe, 4 cases of wedge resection in lower lobe), in 3 patient underwent wedge resection in upper lobe on post-operative day 1 and no patient on post-operative day 30. The mean size of residual pneumothorax was 1.3 cm (0.8–2.1). Pleural effusion which required drainage was noted in 1 patient in each group. Mild subcutaneous emphysema was noted and absorbed gradually in 9 patients.

The postoperative VAS score was significantly lower in tubeless group than in drainage group in post-operative day 1 and 3. Side effects were rare and mild, including cough and hemoptysis. Unsatisfactory wound healing was less in tubeless group, however, there was no significant difference. No re-intervention or readmission occurred.

## Discussion

The result of this research indicates that thoracoscopic wedge resection for peripheral lung nodules without chest tube drainage is safe, all air leaks have been adequately handled before wound closure. Patients in tubeless group have less postoperative pain and shorter hospital stay compared to patients in drainage group. Although not significantly different, would healing in tubeless group is better.

The concerns associated with omitting chest tube drainage after pulmonary resection refer to the risk of pneumothorax, bleeding, and pleural effusions [5]. Risk of large pneumothorax, symptomatic bleeding, and effusions is low in patients with normal pulmonary function who underwent wedge resection [6].

The reported incidence rate of pneumothorax was about 40% at 6 h and 1 day post-operatively, 6.6% on day 14 in previous study in tubeless thoracoscopic surgery [3]. It seemed stable and safe, however, pneumothorax might still affect the mechanics of the lung and lead to reductions in lung compliance, vital capacity, total capacity, and functional residual capacity, besides, complications of pneumothorax might occur, including subcutaneous emphysema, pleural effusion, or even pneumomediastinum [7].

In this series, traditional water-seal leakage test was applied, then a 22–24F chest tube was used to further test for existence of air leak, it also showed good property for drainage of air in a proper position. When patients in reverse trendelenburg position with 30°, complete air drainage is more easily to be achieved by a chest tube. The observation also suggests that residual air following surgery could be absorbed safely and quickly. In tubeless group with modified air leak test and chest tube drainage, pneumothorax was rare and mild with low incidence, patients recovered better.

With the application of tubeless approach, this method resulted in relief of symptoms, low rate of complications and fast recovery [8]. Previous studies showed that patients who underwent non-intubated surgery correlated with shorter postoperative hospital stays and postoperative fasting time, they also exhibited a trend toward lower cardiovascular complication rates (1.45% vs 2.69%) and respiratory complication rates (8.23% vs 11.18%) [9, 10]. Compared with multiportal VATS, uniportal VATS might reduce post-operation pain and lead to faster recovery [11]. Uni-portal VATS only invades a single intercostal region, thoracic muscles could also be spared, leading to less intercostal nerve disorder and less postoperative pain [12]. Patients underwent uni-portal VATS usually had less pain, chronic pain syndrome or shoulder dysfunction was rare.

VAS scores in tubeless group were lower than drainage group in POD 1 and 3, they were comparable in POD 7, 30 and 60, which indicated that postoperative pain was mainly caused by chest tube placement [13, 14]. Complications were rare in this series, pain was mild, patients in tubeless group might recover better.

Thoracoscopic surgery without endotracheal intubation could avoid intubation and mechanical ventilation-related side effects [15]. Several studies showed the technical feasibility and safety of this technique, complication rates was lower, including sore throat, nausea, irritable cough, urinary retention, with shorter length of hospital stay [16]. Hypercapnia might occur during operation, a laryngeal mask coulds be used to maintain satisfactory oxygenation, preventing gastric reflux and aspiration [17, 18]. Spontaneous ventilation could also be assisted by hand thorough laryngeal mask for better lung expansion.

There were several limitations to this present study. For retrospective nature of the study, the randomization was absent, and selection bias cannot be eliminated. Although tubeless thoracoscopic wedge resection seemed to have better peri-operative result, it may be partially related to the biases in the selection and evaluation of patients for the tubeless approach, determination of postoperative pain scores was also not blinded either for the patient or for the treating physician. Prospective research was needed to further confirm the conclusion. Small sample size and short follow-up time were also the main limitations, long-term and subjective patient outcomes should be established in future studies to assess both peri-operative outcome and oncologic efficacy. Besides, further investigation was needed to prove whether tubeless technique was fit for more complicated operation (lobectomy or segmentectomy).

## Conclusions

Tubeless uniportal thoracoscopic wedge resection with modified air leak test and chest tube drainage is feasible and safe for selected patients with peripheral pulmonary nodules, it might reduce post-operation pain and lead to faster recovery.

## Data Availability

The datasets used and/or analysed during the current study are available from the corresponding author on reasonable request.

## Abbreviations

VATS: Video-assisted thoracoscopic surgery
PPN: Peripheral pulmonary nodules
GGO: Ground glass opacity
ASA: American Society of Anaesthesiologists
CT: Computer tomography
TCI: Target-controlled infusion
VAS: Visual Analogue Scale
FEV1: Forced expiratory volume in 1 s
BMI: Body mass index
SpO_2_: Saturation of peripheral oxygen
EtCO_2_: End-tidal carbon dioxide
MIA: Minimal invasive adenocarcinoma
IAC: Invasive adenocarcinoma
AIS: Adenocarcinoma in situ
AAH: Atypical adenomatous hyperplasia
PSM: Propensity score match
